# A Case Report of Treatment With Cariprazine in a Recurrent Psychosis Presumably Induced by Methamphetamine

**DOI:** 10.7759/cureus.47135

**Published:** 2023-10-16

**Authors:** Robert A Moran, Cydne Hastings, Una Della-Pietra, Chinar Singh, Mario Jacome

**Affiliations:** 1 Department of Psychiatry, Family Center for Recovery, Lantana, USA; 2 College of Osteopathic Medicine, Nova Southeastern University Dr. Kiran C. Patel College of Osteopathic Medicine, Fort Lauderdale, USA; 3 Department of Osteopathic Medicine, Nova Southeastern University Dr. Kiran C. Patel College of Osteopathic Medicine, Fort Lauderdale, USA

**Keywords:** substance use disorder (sud), cariprazine, mesocorticolimbic pathway, methamphetamine-associated psychosis, methamphetamine use

## Abstract

This case report describes a 31-year-old male patient with psychosis presumably related to methamphetamine-associated psychosis (MAP). Our patient was experiencing persistent symptoms of visual, tactile, and auditory hallucinations after cessation of methamphetamine. He has a medical history of a substance use disorder, post-traumatic stress disorder, attention-deficit hyperactivity disorder, nicotine dependence and major depressive disorder. Previously, he received a wide range of antipsychotic drug treatment regimens at other psychiatric facilities, all with some degree of effect, but never with complete symptom relief. At the time of admission to our inpatient clinic, he was started on cariprazine and reported a significant decrease in visual, auditory, and tactile hallucinations with complete cessation for a period of two weeks. There appears to be a unique ability of cariprazine’s mechanism of action to reverse symptoms of the presumable diagnosis of MAP that is unable to be achieved with other antipsychotic medications.

## Introduction

Methamphetamine (MA) is a highly lipophilic molecule that penetrates the blood-brain barrier and alters noradrenergic, serotonergic, and dopaminergic pathways [1]. Dopamine acts on the central nervous system (CNS) via three pathways, namely the mesolimbic, the mesocortical, and the nigrostriatal pathways [2-12]. MA is typically associated with dopaminergic neurotransmission involving the mesocorticolimbic pathway [3-12]. With repeated use of MA, the release of dopamine in the mesolimbic pathway increases, in turn, leading to reinforcement of behavior [1]. There is also an increase in the number of the D3 receptors which bind with high affinity to dopamine [4]. This combined effect may lead to addiction and increased cravings, often requiring users to increase dosages of MA for sustained effects [4].

MA, being an addictive and potent psychostimulant, can produce a transient psychotic state termed “methamphetamine-associated psychosis” (MAP) [4]. It is estimated that 40% of MA users will experience psychotic symptoms such as tactile, visual, and auditory hallucinations, ideas of reference, and paranoid delusions [5-14]. It has been reported that compared to the general population, MA users are 11 times more likely to develop psychosis [6]. MAP typically disappears after an acute phase but in some susceptible individuals may go on to persist for many months or even years after abstinence from the drug [5-13]. Because MAP can present as a persistent psychotic syndrome that closely mimics paranoid schizophrenia, many clinicians find it challenging to differentiate between the two diseases and assign a correct diagnosis [3-14]. Reports have shown that 15-28% of patients admitted to the hospital for MAP need hospitalization following admission for more than 2-3 months due to persistent psychosis [3]. Typically, 10-28% of patients with MAP display psychosis for more than six months following MA cessation, whereas 28% of MA users tend to show “schizophrenia-like symptoms” for an average of 8-12 years following abstinence of the drug [3]. One potential theory for these persistent symptoms considers secondary chronic hyperactivity of the mesolimbic dopaminergic pathway to explain the recurrent psychotic symptoms after the patient has quit the drug.

MAP is typically treated with a multidisciplinary approach that includes the use of antipsychotic medications. One of these medications, cariprazine, currently most known for its ability to treat negative symptoms of schizophrenia, has recently gained recognition in effectively treating MAP as well. Cariprazine has a unique pharmacodynamic profile among the class of antipsychotics. It is a partial agonist that acts uniquely with greater affinity and selectivity for the D3 receptor compared to other antipsychotics [4]. Partial agonism is also evident at the D2, 5HT1A, 5HT1B, and 5HT1 in dose-dependent manners [4]. Due to this unique biochemical profile with marked activity on the D3 receptor, it has shown promise for the future of decreasing psychotic symptoms in patients with MAP [4]. This case study outlines a patient with presumable MAP involving symptoms of visual, auditory, and tactile hallucinations managed using cariprazine.

## Case presentation

A 31-year-old Hispanic male presented to an inpatient mental health treatment program following an acute psychotic episode with prior comorbid substance use. He was seeking treatment and detoxification from MA and cannabis. On initial presentation, the patient was actively experiencing visual, auditory, and tactile hallucinations without insight. Per the patient, he has a medical history of substance use disorder, post-traumatic stress disorder (PTSD), attention-deficit hyperactivity disorder (ADHD), and nicotine dependence.

Background medical history

A chronological review of his past medical records indicated he was diagnosed with PTSD at the age of 10 following a sexual assault. At 11 years old, he was diagnosed with ADHD due to poor impulse control and hyperactivity and prescribed methylphenidate daily. Furthermore, he started using cannabis at the age of 15 which progressed to 3,4-Methylenedioxymethamphetamine (MDMA) and cocaine use when he was 18 years old. He reported that this was the first time he experienced hallucinations. Furthermore, when he was 21 years old, he had a knee arthroscopy which led to misuse of opiates. From the age of 25 to 29 years old, the patient reported engaging in recreational cannabis, alcohol, fentanyl, phencyclidine, heroin, psilocybin mushrooms, MA, ketamine, and gamma-hydroxybutyrate (GHB).

Additionally, the patient was involuntarily institutionalized under the Baker Act at 29 years of age for suicidal ideation in March of 2022. After discharge from the hospital, he received treatment through an outpatient psychiatric clinic for two months and at this time he remained substance-free. Despite the discontinuation of all recreational drug use, the patient started to complain about intermittent visual, tactile, and auditory hallucinations. His psychiatrist diagnosed him with schizophrenia and general anxiety disorder (GAD). He was prescribed olanzapine, fluoxetine, clonidine, clonazepam, buprenorphine, and aripiprazole. Due to his persistent psychosis, his psychiatrist also prescribed cariprazine 1.5mg daily. Shortly after, he relapsed on MA and cannabis.

Current psychotic episode

Due to insufficient outpatient treatment and current use of only cannabis and MA, he sought out residential inpatient treatment with our facility at the age of 30 in May 2022. Upon intake, he was actively experiencing visual, auditory, and tactile hallucinations with poor insight and cognitive dysfunction. The patient reported he has been intermittently hallucinating since his relapse. The adverse effects of MA are unpredictable and in some cases may cause a psychotic break that can persist beyond the period of intoxication. Consequently, he was medically detoxed from cannabis and MA at our facility at this time. His treatment plan included individual behavioral therapy and group therapy to prevent the resumption of MA use and pharmacologic treatment with an increased dose of cariprazine which was titrated to 6mg daily. Two weeks later, the patient reported that his hallucinations “decreased to 60%” but the hallucinations weren’t completely gone. MA use can trigger psychosis in patients who have previously experienced psychotic symptoms for years and has been termed the “sensitization” effect [5].

Due to the decrease in hallucinations, several interviewing approaches were conducted to accurately optimize the differential diagnosis of substance-induced versus primary psychiatric disorders (e.g. schizophrenia). Interviewing processes were used such as the Psychiatric Research Interview for Substance and Mental Disorders (PRISM), which has excellent reliability for psychotic disorder diagnoses among individuals with alcohol and/or drug use disorders [5]. This was particularly helpful with our evaluating patient, an MA user who presented with psychosis, because MA users may suffer from a recurrence of psychotic symptoms despite complete drug detoxification. With the PRISM method, the interviewer takes into consideration the patient's timeline of all drug use, periods of substances and abstinence, previous treatments, and major life events. According to this method and the Diagnostic and Statistical Manual of Mental Disorders (DMS-IV) criteria, if psychotic symptoms occur entirely during a period of drug use or withdrawal and are more severe then a substance-induced psychosis is diagnosed [5].

Furthermore, the DSM-V states that an episode of psychosis that occurs in the context of MA (or other substance) use can be considered a primary psychotic disorder (e.g. schizophrenia) if there is a past history of psychotic episodes that are not substance-related or psychotic symptoms occur at least one month after cessation of acute withdrawal [5] which was not the case in our patient. In contrast, substance-induced psychotic disorders are diagnosed as hallucinations or delusions developing soon after, during withdrawal or intoxication from a substance that is known to cause such psychotic symptoms and the presence of noticeable delusions or hallucinations. Accordingly, the diagnosis of MAP can be determined when the recognizable psychotic symptoms overtake the effects that are known to be seen in withdrawal and intoxication of MA [5].

Administration of the positive and negative syndrome scale (PANSS) was then performed to provide an objective scoring of his schizophrenia-like symptoms. In the span of one month, his score decreased from a total score of 73, categorized as mildly ill, to 46, categorized as normal. PANSS includes a mathematical score that is used to calculate a response to an intervention [15,16].

Objective diagnostic instruments were also used such as the Composite International Diagnostic Interview (CIDI) and Diagnostic Interview Schedule (DIS) from the DSM-V which are structural interview approaches that rely on clinical judgment viewing the presentation of the etiology of psychotic symptoms in regards to substance misuse. A combination of the outcome and results of these scales, clinical judgment, DSM-IV and V criteria, and prior history along with a significant reduction of his symptoms with the dose of cariprazine led to a diagnosis change from schizophrenia to unspecified psychosis likely due to MAP.

The patient left against medical advice (AMA) a month after his initial admission into our treatment program and relapsed on MA, fentanyl, cannabis, N,N-Dimethyltryptamine (DMT), GHB, and heroin. He was then arrested for grand theft, placed under the Marchman Act, and readmitted to our facility. At our facility, he went through detoxification once more. He reported being off cariprazine during the time of his relapse and still experienced tactile hallucinations of spiders crawling on him, visual hallucinations of being stabbed, and olfactory hallucinations of burnt wood. His cariprazine dose was titrated over the next two months reaching a max dose of 10 mg daily due to persistent hallucinations. The patient reported that with the dose change, “the hallucinations come and go; I know they’re not real, and I don’t know when I will hallucinate next.” During these episodes, the hallucinations became typically visual in nature with infrequent episodes containing a combination of tactile, visual, and olfactory hallucinations further supporting our diagnosis and treatment plan. Unfortunately, the patient left the facility three months later. Therefore, we were unable to further continue the trial of cariprazine but we do believe future studies on the use of cariprazine and MAP should show promises of remission.

## Discussion

Our patient experienced new-onset hallucinations following the use of MA. At a dose of 10 mg of cariprazine, he reported a significant decrease in visual, auditory, and tactile hallucinations with complete cessation for a period of two weeks. There are several case reports demonstrating MAP with cariprazine producing a therapeutic response. Ricci et al. describe a case with complete cessation of persistent psychosis secondary to MA use only after initiation of cariprazine, with prior antipsychotic trials failing to decrease symptoms [4].

Additionally, Truong et al. reported a case of cessation of MA cravings, hypervigilance, and paranoia that was exacerbated by prior MA use after one-month treatment of 1.5mg daily cariprazine [7]. Finally, Cruz et al. describe a patient with schizophrenia and comorbid MA use disorder that found a significant decrease in demand for substance use and improvement in cognitive function with a combination of quetiapine and cariprazine [8]. There appears to be a unique ability of cariprazine’s mechanism of action to reverse symptoms of substance-induced psychosis as a result of MA that is unable to be achieved with other antipsychotic medications. While this may be due to minimal reporting of MAP treatment in literature combined with the variability of patient metabolism and drug response, it is also possible that cariprazine may have a unique mechanism that makes it more suitable in treatment for patients with MAP due to the brain changes that occur following MA use.

Recurrent psychotic symptoms following MA cessation are speculated to be related to secondary hyperactivity of the mesolimbic dopaminergic pathway. This further elucidates that dopamine receptor antagonists such as cariprazine are effective during the abstinence of the drug [6]. Cariprazine’s mechanism of action is most notable for its D3 activity. Compared to aripiprazole, cariprazine has a 3- to 10-fold greater affinity for the D3 receptor [9]. Additionally, cariprazine binds to D3 receptors with higher affinity than dopamine itself, obstructing the binding of the D3 receptors with the excess dopamine made available in the mesolimbic pathway after increased MA use [4-7].

Additionally, studies have shown a positive correlation between childhood abuse with a high risk of MAP and substance use disorders [17]. Ding et al. conducted a cross-sectional study among MA users currently in rehabilitation who experienced adverse childhood experiences such as sexual/physical abuse which could be a potential correlation seen in our patient [18]. Additional risk factors for MAP have also been studied in Taiwanese, Japanese, and Australian populations and include a family history of the condition, other substance use disorders, or a comorbid personality or mood disorder [19]. Further research is needed on other potential risk factors, especially in U.S. populations, including genetic differences, that may make an individual susceptible to MAP.

Furthermore, there is evidence of reduction of frontotemporal cortical thickness in patients with MAP and schizophrenia demonstrating similar neuropathological and glial changes in the frontal cortex inducing severe positive symptoms. A study conducted by Ulhmann et al. on MAP demonstrated a reduction in hippocampal volume and thinner frontotemporal cortical thickness of the brain when compared to control groups [10]. In another study conducted by Jia et al., researchers compared gray matter volumes in control patients, patients with schizophrenia, MA users without psychosis, and patients with MAP [6]. Results showed a similar reduction in gray matter reduction between the MAP group and the schizophrenia group in the prefrontal cortex. In addition, a study conducted by Aoki et al. investigated gray matter reductions using neuroimaging [11]. Analysis of neuroimaging showed substantial gray matter volume reductions in left perisylvian structures essentially the anterior superior temporal gyrus, posterior inferior frontal gyrus, frontopolar cortices, and white matter reduction compared to controls. In the patients with MAP, the medial portion of the frontopolar cortex was substantially correlated with severe positive symptoms. These results demonstrated supplementary evidence that MAP and schizophrenia show similar neuropathological changes making it difficult to differentiate between the two diagnoses.

Some potential limitation of our case is the possibility of new-onset hallucinations as a progression of our patient’s chronic episodes. However, given our patient’s established history with no prior hallucinations before the use of MA, it is likely these hallucinations were a result of the drug although it is difficult to say since he has a history of polysubstance use. This leads us to question whether this was the result of a sole trigger or a combination of substances. Additionally, another common limitation could be present on the CIDI and DIS because it's dependent on self-reporting therefore this needs to be taken into consideration. Furthermore, according to multiple systematic reviews that have been performed in the past, there seems to be a discrepancy in the calculation of the PANSS scores and a lack of standardization of the method of calculation. Therefore, although the PANSS scoring for our patient shows improvement, there is a possibility that the improvement is an overestimation and a false positive classification. In addition, we were unable to follow up with this patient which serves as another limitation to our study.

## Conclusions

MA use resulting in hallucinations is a seemingly rare phenomenon. Overall, the use of cariprazine for MAP should be a consideration in patients with persistent hallucinations despite abstinence from MA. The improvements noted in our patient with increasing dosage of cariprazine show a correlation between the use of this medication and symptoms of MAP. Further research is critical for helping distinguish the differences between MAP and schizophrenia in order to create a larger MAP sample size for future studies, as diagnosis of this condition can be challenging. With further expansion of sample size, the potential for individual variability may be lessened, revealing the true therapeutic potential of cariprazine on MAP. Further research is needed on this topic, especially among patients who demonstrate symptoms of psychosis related to substance misuse.
